# Association between Mediterranean Dietary Pattern and Breakfast Quality with Physical Fitness in School Children: The HIIT Project

**DOI:** 10.3390/nu13041353

**Published:** 2021-04-18

**Authors:** Cristina Cadenas-Sanchez, María Medrano, Lide Arenaza, Maria Amasene, Maddi Osés, Idoia Labayen

**Affiliations:** 1 Institute for Innovation & Sustainable Development in Food Chain (IS-FOOD), Public University of Navarra, 31006 Pamplona, Spain; maria.medrano@unavarra.es (M.M.); lide.arenaza@unavarra.es (L.A.); maddi.oses@unavarra.es (M.O.); idoia.labayen@unavarra.es (I.L.); 2 Department of Health Sciences, Public University of Navarra, 31006 Pamplona, Spain; 3 IdiSNA, Navarra Institute for Health Research, 31008 Pamplona, Spain; 4 Department of Pharmacy and Food Science, Faculty of Pharmacy, University of the Basque Country (UPV/EHU), 48940 Vitoria-Gasteiz, Spain; maria.amasene@ehu.eus

**Keywords:** diet quality, cardiorespiratory fitness, muscular strength, speed–agility, physical activity, youth

## Abstract

Dietary habits have been linked with health in childhood. However, few studies have examined the association between healthy dietary patterns and physical fitness. Therefore, the aim of this study was to examine the associations of adherence to the Mediterranean dietary pattern (MDP) and breakfast quality with physical fitness in children. Further to this, we examined the role of physical activity in these associations. A total of 175 children (86 girls, 9.7 ± 0.3 years) participated. Adherence to MDP and breakfast quality were assessed by the KIDMED questionnaire and 24 h recall, respectively. Cardiorespiratory fitness, muscular strength, and speed–agility were assessed. Physical activity was evaluated by wrist-worn accelerometers. Greater adherence to the MDP was related with higher cardiorespiratory fitness, lower-limbs muscular strength, and speed–agility (all β ≥ 0.189, all *p* ≤ 0.02). No significant associations were observed between breakfast quality and physical fitness (all *p* > 0.05). However, all the significant associations disappeared after adjusting for physical activity (all *p* > 0.05). Our study sheds light on the relevance of adhering to the MDP over physical fitness in school children. However, there is no association between breakfast quality and physical fitness. Furthermore, physical activity seems to explain, at least partially, these findings.

## 1. Introduction

Healthy dietary habits are important protective factors of health [1,2]. The Mediterranean dietary pattern (MDP), which is characterized by a high consumption of fruits and vegetables, nuts, cereals, fish, and olive oil, and minimal amounts of red meat and dairy products, has been associated with beneficial effects on health [3]. Indeed, it is known that greater adherence to the MDP may reduce cardiovascular disease risk factors and mortality [1,2]. In children, high adherence to the MDP is associated with lower obesity at the cross-sectional level [4,5], and protection against adiposity gain 2 years later [6]. The quality of breakfast is also very relevant when considering healthy dietary habits. Breakfast is the first meal of the day and breaks up the long period of fasting, being considered as the most important meal of the day [7]. In Spain, breakfast is predominantly composed of sugary and fat-rich products (e.g., pastries, cereals, artificial juices, milkshakes, etc.) which might affect the glycaemia, dyslipidaemia, and appetite of children [8,9]. Thereby, having healthy dietary patterns, such as a good adherence to the MDP and a high quality breakfast, are of great interest and concern over youth health.

Together with dietary habits, physical fitness, particularly cardiorespiratory fitness, has been widely recognised over the last two decades as a powerful marker of health in children and adolescents [10,11]. Cardiorespiratory fitness, which refers to the capacity of the circulatory, respiratory, and muscular systems to supply oxygen for energy production needed during physical activity, has been shown to be an important predictor of cardiometabolic disorders and premature mortality later in life [10,11]. Therefore, the importance of being fit is essential to prevent health problems already in childhood. 

At early ages, it seems that combining healthy dietary habits, good physical fitness, and daily practice of physical activity are the cornerstone to present and future healthy lifestyle [10,11,12,13]. Although they are independent lifestyle factors, they do not act in isolation and the effects of multiple healthy lifestyle behaviours might be greater than the individual impact [14]. On this line, a recent systematic review and meta-analysis synthesized the evidence regarding how the MDP contributes to acquiring healthy habits, including physical fitness (i.e., cardiorespiratory fitness, muscular strength, and speed–agility) [15]. The authors concluded that higher adherence to the MDP was significantly associated with better cardiorespiratory fitness, muscular strength, and speed–agility in children and adolescents [15]. With regard to breakfast quality, no studies were found examining its association with physical fitness components. Thus, further studies examining the association between dietary patterns and physical fitness in children are needed.

Therefore, the aim of this study was to explore the associations of MDP and breakfast quality with physical fitness in school children. Furthermore, we examined whether physical activity modifies these associations among children.

## 2. Materials and Methods

### 2.1. Study Design and Participants

This study is under the framework of the High Intensity Interval Training (HIIT) project (Clinical trial reference number: NCT03479658), a randomized controlled trial that aims to investigate the effects of different doses of 8 weeks of HIIT on body composition, physical fitness, and physical activity in 9–10-year-old children. This work uses baseline data collected prior to randomization. Children were recruited from two different schools in Navarra (Spain). 

A total of 175 school children (50.3% girls, mean age 9.7 ± 0.3 years) were included in this study (convenience sampling). The inclusion criteria were being 9–10-years-old and not having psychological or physical disorders that could hamper exercising and evaluations. Parents or legal guardians were informed about the aims, design, and protocol of the study. If they agreed, they signed the informed consent in order to participate in the study. The study followed the ethical guidelines of the Declaration of Helsinki (1964; revised version in 2013), and was approved by the Ethics Committee of the Public University of Navarra. 

We performed statistical power calculations using the G*Power software (version 3.1.9.2) [16]. Our sample size is sufficient to detect small association sizes (f^2^ ≤ 0.08) considering a statistical power of 80% and an alpha error of 0.05.

### 2.2. Measurements

#### 2.2.1. Body Composition

Weight (kg), percentage of body fat, and body fat-free mass (kg) of participants were measured by bioimpedance (Tanita SC240-MA, Amsterdam, The Netherlands). Height (cm) was measured with a stadiometer (SECA model, Hamburg, Germany). All the body composition assessments were performed in underwear and without shoes. Body mass index (BMI) was calculated as weight divided by height squared (kg/m^2^). Weight status (normal weight, overweight, and obesity) was classified based on the sex- and age-specific BMI cut-off points provided by the World Obesity Federation [17]. All measurements were taken twice, and the mean value was recorded for analyses.

#### 2.2.2. Mediterranean Dietary Pattern

Adherence to the MDP was evaluated using the KIDMED questionnaire (Mediterranean Diet Quality Index for children and teenagers) which is a valid and widely used assessment tool in children [18]. The KIDMED questionnaire is composed of 16 nutritional items: 10 questions about the consumption of diverse food groups and 6 about healthy dietary habits not directly related to the MDP [6]. For the analyses, only those 10 questions related with the MDP were considered. One point was given to the questions related to MDP. Then, the MDP score ranged from 0 (lowest adherence to the MDP) to 10 (highest adherence to the MDP).

#### 2.2.3. Breakfast Quality

One 24 h recall of a weekday or weekend day was recorded by trained nutritionists. Children described their intake from the previous day in an interview. To estimate servings and food sizes, food servings pictures were used [19]. Specifically, the quality of the breakfast was evaluated based on the modified tool “Breakfast Quality Index” (BQI) [20]. In our study, the index used is composed of 10 items related to breakfast intake. The criteria to score one point in each item are: to eat (1) cereals and derivates, (2) fruits and/or vegetables, (3) dairy products, (4) to provide <5% of total daily energy from food rich in simple sugars from breakfast, (5) to consume olive oil, (6) monounsaturated to saturated fatty acids ratio ≥2:1, (7) to provide 20–25% of energy from breakfast in relation to total daily energy intake, (8) to consume cereals, fruits, and dairy products at the same meal, (9) to consume ≥200–300 mg of calcium, and (10) not to consume butter or margarine. Therefore, the BQI score ranged from 0 (poorest breakfast quality) to 10 (optimal breakfast quality).

#### 2.2.4. Physical Fitness

Physical fitness was assessed by the Assessing Levels of PHysical Activity (ALPHA) battery, which is known to be feasible, reliable and valid in children [21].

Cardiorespiratory fitness was evaluated by the 20 m shuttle run test [22]. Briefly, the test consisted of running a 20 m distance following a beep signal. The initial speed was 8.5 km/h (0.5 km/h/min increment). The test finished when the children stopped due to fatigue or failure to reach the end lines concurrent with the audio signal on two consecutive occasions. The number of laps performed was used for the analyses.

Upper-limbs muscular strength was measured by handgrip strength (kg) test which consisted of squeezing the digital dynamometer (TKK 5401, Grip-D, Takei, Tokyo, Japan) as much as possible for 2–3 s. Each hand was measured twice, and the best result of each hand was retained and averaged for the analyses. Lower-limbs muscular strength was measured by standing broad jump test (cm). This test consisted of jumping as far as possible remaining upright. The distance jumped from the start line to the back of the heel (the nearest point of contact when landing) was recorded. The test was performed twice, and the maximum value was used for the analyses.

Speed–agility was assessed by the 4 × 10 m shuttle run test (seconds). This test consisted of running back and forth, 10 m apart, as fast as possible. The children had to exchange sponges at each line. This test was performed two times, and the fastest completion time was recorded for analyses.

#### 2.2.5. Physical Activity

Time spent in physical activity was registered by accelerometers (GT3X+, ActiGraph, Pensacola, FL, USA). Children wore an accelerometer placed on the non-dominant wrist during 7 consecutive days (24 h). They were instructed to remove the accelerometers only for water activities (i.e., bathing or swimming). For the analyses, valid data were considered when the children recorded ≥ 600 min/day of waking hours, and a minimum of 3 days (2 weekdays and 1 weekend day) [23]. Non-wear time was detected using a 20 min time window of consecutive zero counts. The epoch was set at 15 s using the normal filter. Moderate-to-vigorous physical activity was calculated using the cut-off established by Chandler et al. [24]. All data collected at a sampling frequency of 100 Hz were processed using ActiLife software (v.6, ActiGraph, Pensacola, FL, USA).

### 2.3. Statistical Analyses

The characteristics of the study sample were examined using descriptive (mean and standard deviations (SD)) or frequencies analyses (N, %). Confounders were selected after examining their role in the association of MDP and breakfast quality estimates with physical fitness components. Therefore, sex and age were included as basic confounders. For sensitivity analysis, we additionally included school as a covariate.

To investigate relationships between predictors (i.e., MDP and breakfast quality) and outcomes (i.e., physical fitness components), each outcome was regressed on each predictor, adjusting for the basic confounders of age and sex (Model 1). Additional analyses were adjusted for moderate-to-vigorous physical activity and accelerometer awake wearing time (Model 2). As exploratory analysis, we further observed the relationship between physical fitness and physical activity outcomes.

Analyses of covariance were performed using MDP and breakfast quality level (low vs. high) as fixed factors and physical fitness components (i.e., cardiorespiratory fitness, upper- and lower-limbs muscular strength, and speed–agility) as dependent variables. Analyses were adjusted for basic confounders (Model 1). In Model 2, we additionally adjusted for physical activity (i.e., moderate-to-vigorous physical activity and accelerometer awake wearing time). Regarding the MDP index, scores ≥ 7 were classified as average to optimal adherence (named as high adherence) to the MDP [18], and according to the BQI, scores of ≥6 were categorized as medium to high breakfast quality (named as high quality) [20].

All the analyses were performed using SPSS statistics version 22 (IBM Corporation, New York, NY, USA). The level of significance was set at *p* < 0.05.

## 3. Results

Descriptive characteristics (i.e., sociodemographic, adherence to the MDP, breakfast quality index, physical fitness, and physical activity) of the study sample are shown in Table 1. The number of participants vary among measurements (i.e., missing data) due to the following reasons: (i) did not attend school (*n* = 2), (ii) did not have complete or valid data on MDP (*n* = 45), and (iii) did not wear the accelerometer or did not have valid data (*n* = 14).

Table 2 shows the coefficients from the linear regression of each of the physical fitness measures on each predictor (MDP and breakfast quality) after adjusting for basic confounders (sex and age; Model 1) and after additionally adjusting for physical activity confounders (moderate-to-vigorous physical activity and accelerometer wearing time; Model 2). Cardiorespiratory fitness, lower-limbs muscular strength and speed–agility were associated with higher adherence to the MDP (all β ≥ 0.189, all *p* ≤ 0.02) after adjusting for age and sex. No significant relationships were observed between any of the examined physical fitness components and breakfast quality index (all β ≤ 0.115, all *p* ≥ 0.1). When the analyses were additionally adjusted for physical activity (Model 2), all the results became statistically non-significant (all β ≤ 0.160, all *p* ≥ 0.07). Relationships between breakfast quality and physical fitness components remained non-significant after adjusting for age, sex, moderate-to-vigorous physical activity and accelerometer wearing time (all β ≤ 0.093, all *p* ≥ 0.2). Supplementary analysis shows that cardiorespiratory fitness, lower-limbs muscular strength and speed–agility were associated with moderate-to-vigorous physical activity (Table S1, all r ≥ 0.245, *p* < 0.01).

**Figure 1:**
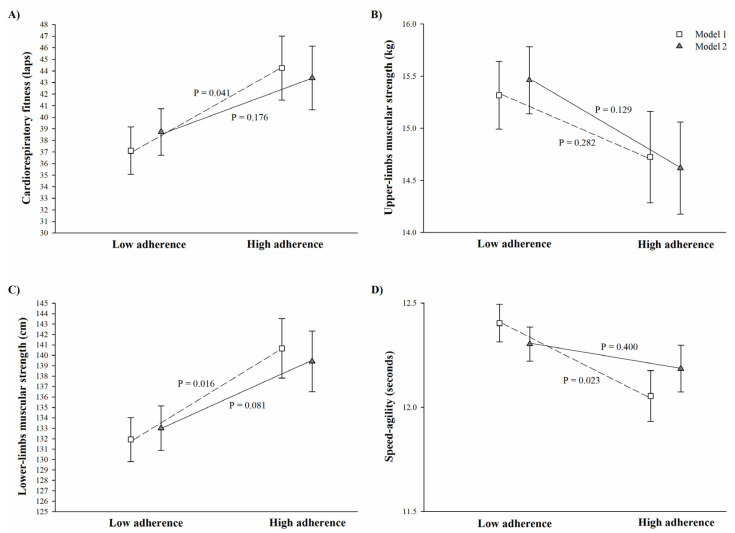
Differences between low and high adherence to the Mediterranean dietary pattern and physical fitness components in children (**Panel A**: cardiorespiratory fitness, **Panel B**: upper-limbs muscular strength, **Panel C**: lower-limbs muscular strength, and **Panel D**: speed–agility).

**Figure 2:**
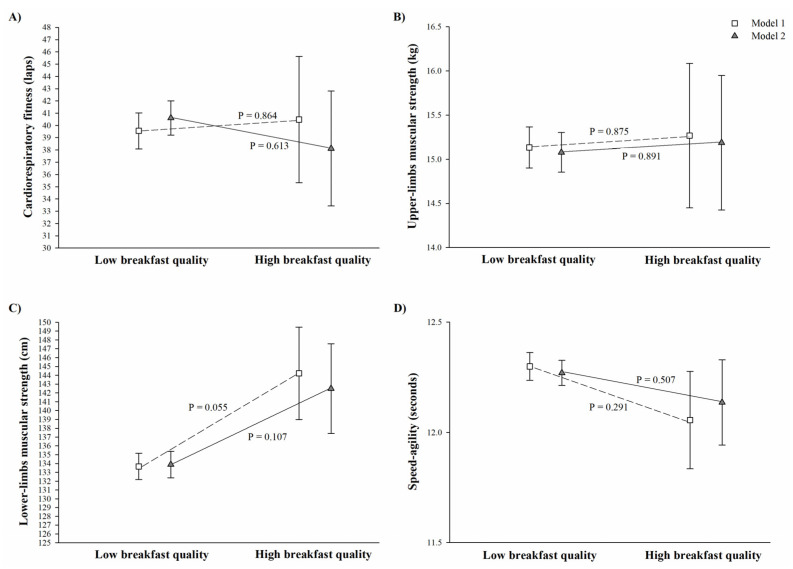
Differences between low and high breakfast quality and physical fitness components in children (**Panel A**: cardiorespiratory fitness, **Panel B**: upper-limbs muscular strength, **Panel C**: lower-limbs muscular strength, and **Panel D**: speed–agility).

Figure 1 depicts the analysis of covariance between adherence to the MDP and physical fitness components. In Model 1, those children grouped in the high adherence to the MDP group presented better cardiorespiratory fitness (44.2 ± 2.8 vs. 37.1 ± 2.0 laps, *p* = 0.041), lower-limb muscular strength (140.7 ± 2.9 vs. 131.9 ± 140.7 cm, *p* = 0.016), and speed–agility (12.05 ± 0.1 vs. 12.4 ± 1.0 s, *p* = 0.023) compared to their peers classified as having low adherence to the MDP (Figure 1). However, after adjusting for physical activity, these differences disappeared (Figure 1, Model 2, all *p* > 0.08). Figure 2, which depicts the analysis of covariance between breakfast quality and physical fitness components, shows that there were no significant difference in physical fitness components between children having low and high quality breakfast in any of the models examined (all *p* > 0.05).

Data are presented adjusted by basic confounders (Model 1, age and sex; represented as empty square), and additionally adjusted for moderate-to-vigorous physical activity and wearing time (Model 2; represented as grey triangle). Classification of adherence to the Mediterranean dietary pattern was based on a cut-off score of 7 on the 10 used questions of the KIDMED questionnaire; the high adherence group includes those participants classified as having average to optimal adherence to the Mediterranean dietary pattern. Sample size varied for Model 1 (n low adherence = 84, and n high adherence = 46) and Model 2 (n low adherence = 78, and n high adherence = 42). In the speed–agility test, less time indicated a faster runner; therefore, a lower score indicates better performance.

Data are presented adjusted by basic confounders (Model 1, age and sex; represented as empty square), and additionally adjusted for moderate-to-vigorous physical activity and wearing time (Model 2; represented as grey triangle). Classification of low and high breakfast quality was based on a cut-off score of 6 on the 10 items; the high breakfast quality group includes those participants classified as medium to high breakfast quality. Sample size varied for Model 1 (n low breakfast quality = 158, and n high breakfast quality = 13) and Model 2 (n low breakfast quality = 144, and n high breakfast quality = 13). In the speed–agility test, less time indicated a faster runner; therefore, a lower score indicates better performance.

## 4. Discussion

The main finding of this study is that higher adherence to the MDP was associated with better cardiorespiratory fitness, lower-limbs muscular strength and speed–agility; however, after adjusting for physical activity levels, these results were attenuated, showing the role of moderate-to-vigorous physical activity on these associations. In addition, our results also showed no significant relationship between the breakfast quality index and any of the physical fitness components in children.

### 4.1. Mediterranean Dietary Pattern, Breakfast Quality, and Physical Fitness

In the last few years, a growing body of studies have reported the influence of dietary patterns on children and adolescents’ health. Yet, less is known about the association between dietary patterns and physical fitness, which is an important marker of cardiovascular health even in childhood. In our study, we observed that children with greater adherence to the MDP showed higher cardiorespiratory fitness, lower-limbs muscular strength, and speed–agility. Moreover, those who were classified into the high adherence MDP group also showed better cardiorespiratory fitness (19%), muscular strength (7%), and speed–agility (3%) levels compared to those who were classified as the low adherence MDP group. Our findings are in accordance with a recent meta-analysis where the authors observed that MDP was positively related to cardiorespiratory fitness, muscular strength, and speed–agility in children and adolescents from 3 to 18 years (n = 13 unique studies related with fitness; standardized mean difference range from 0.06 to 0.22, *p* < 0.05) [15]. The observed positive findings could be explained by the characteristics of the MDP intake (e.g., high consumption in fruits, vegetables, nuts, cereals, fish, etc.) which could yield benefits on physical fitness in these early ages. However, longitudinal studies examining the influence of the MDP on fitness over time, as well as randomized controlled trials examining the effect of improving the adherence to the MDP on changes regarding fitness levels are needed to elucidate the direction and causality of our findings.

In regard to breakfast quality, our findings showed no significant association with any of the examined physical fitness components. To the best of our knowledge, there are no previous studies examining the association of these variables in youth, which hampers any comparison. However, our hypothesis on the absence of association is that the breakfast quality index is based on only one meal of the day that could have impacted on children within the first part of the day, but not over the whole day [25,26]. It is important to highlight that most of the information previously provided regarding the influence of breakfast on health parameters or academic performance was focused on breakfast consumption/skipping, but not on breakfast quality, which is also relevant.

### 4.2. Role of Physical Activity on the Association of Dietary Patterns with Physical Fitness

In order to examine the role of physical activity on the association of dietary patterns with physical fitness, we further adjusted the analyses for moderate-to-vigorous physical activity. Our findings depicted that physical activity played an important role on the association of MDP and, to a lesser extent, on breakfast quality with physical fitness components (i.e., significant findings disappeared). These results suggest that those children who eat properly are also those who are more physically active. This is in line with a recent meta-analysis in youth, where Garcia-Hermoso et al. [15] observed that the adherence to the MDP was positively related to physical activity levels (n = 28 unique studies related with fitness; standardized mean difference of 0.14, *p* < 0.05). In line with this assumption, a randomized controlled trial in 8–12-year-old children with overweight/obesity concluded that after a 22-week family-based healthy lifestyle education program, children improved the quality of their diet as well as their cardiometabolic health, and that obesity prevention programs should focus on the avoidance of sugar-sweetened beverages and the promotion of physical activity [9]. Therefore, although more studies examining the role of physical activity on dietary patterns are needed, specifically focused on breakfast quality, it seems that practicing physical activity, mainly activities at high intensity, is positively related to a healthy dietary pattern in youth.

Some practical implications can be derived from this study: (i) improving dietary patterns, mainly through the Mediterranean dietary pattern, could be associated with higher cardiorespiratory fitness, muscular strength, and speed–agility in children; and (ii) schools, parents, and health-related professionals should emphasize the need to promote a healthy lifestyle combining dietary patterns, physical fitness, and physical activity. As an example, a multidisciplinary intervention program focused on improving diet quality, physical fitness and physical activity as well as reducing sedentary time, will contribute to current and future youth health.

This study presents several limitations: (1) its cross-sectional design does not allow for causal inference; and (2) the limited number of 24 h recall (n = 1) performed either on a weekday or weekend days might have limited the general view of overall breakfast quality. However, we have further examined whether differences exist between weekdays and weekends, and no significant difference was observed in regard to the breakfast quality index (*p* = 0.911). The strengths of the present study are: (1) the novelty of the investigation; (2) the use of reliable, valid, and health-related physical fitness measurements; and (3) the use of accelerometers for objectively assessing physical activity.

## 5. Conclusions

Our study sheds light on the influence of adherence to the MDP on physical fitness in school children. However, there is no association between breakfast quality index and physical fitness. Furthermore, physical activity seems to explain, at least partially, these findings. More studies are needed to examine these relationships in children and adolescents to support or contrast our findings. The public health implications resulting from our results highlight the importance of promoting healthy dietary patterns, specifically the MDP pattern’s role in physical fitness in children.

## Figures and Tables

**Table 1:** Descriptive characteristics of the study sample.

		All		Boys		Girls
	N	Mean ± SD	N	Mean ± SD	N	Mean ± SD
Age (years)	173	9.7 ± 0.3	86	9.7 ± 0.3	87	9.6 ± 0.3
Weight (kg)	173	33.2 ± 6.6	86	33.2 ± 7.1	87	33.2 ± 6.1
Height (cm)	173	137.7 ± 5.6	86	137.9 ± 5.8	87	137.4 ± 5.4
Body mass index (kg/m^2^)	173	17.4 ± 2.6	86	17.3 ± 2.7	87	17.5 ± 2.6
Body fat percentage (%)	173	18.7 ± 7.3	86	16.1 ± 5.1	87	21.3 ± 8.2
Fat-free mass (kg)	172	59.4 ± 5.8	85	61.3 ± 3.8	87	57.5 ± 6.8
Weight status (%) *:						
Normal weight	126	72.8%	63	73.3%	63	72.4%
Overweight/Obese	47	27.2%	23	26.7%	24	27.6%
Diet:						
Mediterranean dietary pattern	130	5.9 ± 1.6	63	6.1 ±1.6	67	5.8 ±1.7
Breakfast quality index	175	3.9 ± 1.1	86	3.9 ± 1.2	89	3.8 ± 1.0
Physical fitness:						
Cardiorespiratory fitness (laps)	172	39.5 ± 20.3	84	48.5 ± 21.6	88	31.1 ± 14.6
Upper-limbs muscular strength (kg)	173	15.1 ± 3.0	85	15.7 ± 3.4	88	14.6 ± 2.5
Lower-limbs muscular strength (cm)	172	134.5 ± 19.8	84	141.0 ± 19.4	88	128.2 ± 18.6
Speed–agility (sec)	172	12.3 ± 0.8	84	12.0 ± 0.7	88	12.6 ± 0.8
Physical activity (min/day) ^†^:						
Moderate-to-vigorous physical activity	161	117.6 ± 28.8	79	121.0 ± 27.5	82	114.9 ± 29.6
Wearing time (awake)	161	828.3 ± 41.6	79	831.3 ± 43.1	82	825.2 ± 40.4

SD = Standard deviation. Weight status was presented as frequency and percentage. The rest of the sections were presented as mean ± standard deviation. * Classified according to Cole et al. [17]. ^†^ Classified according to Chandler et al. [24] cut-off points for non-dominant wrist.

**Table 2:** Linear regression analysis between the Mediterranean dietary pattern and breakfast quality with physical fitness components.

	Mediterranean Dietary Pattern			Breakfast Quality Index		
	β	B	*p*	β	B	*p*
Model 1:						
Cardiorespiratory fitness	0.189	2.416	0.019	0.058	1.062	0.406
Upper-limbs muscular strength	−0.089	−0.164	0.312	0.036	0.098	0.628
Lower-limbs muscular strength	0.196	2.542	0.017	0.115	2.058	0.116
Speed–agility *	−0.210	−0.117	0.010	−0.050	−0.038	0.494
Model 2:						
Cardiorespiratory fitness	0.126	1.564	0.116	0.009	0.158	0.898
Upper-limbs muscular strength	−0.160	−0.289	0.071	0.089	0.231	0.247
Lower-limbs muscular strength	0.141	1.796	0.091	0.093	1.695	0.207
Speed–agility *	−0.121	−0.061	0.131	−0.017	−0.012	0.809

Model 1 was adjusted for age and sex. Model 2 was additionally adjusted for moderate-to-vigorous physical activity and accelerometer awake wearing time. Moderate-to-vigorous physical activity was classified according to Chandler et al. [24] cut-off points for non-dominant wrist. β = Beta standardized coefficient. B = Non-standardized beta coefficient. * In the speed–agility test, less time indicates a faster runner, so that a lower score indicates better performance.

## Data Availability

The data presented in this study are available on request from the corresponding author.

## Supplementary Materials

The following are available online at https://www.mdpi.com/article/10.3390/nu13041353/s1, Table S1: Pearson correlation between physical fitness and physical activity.
