# Supplementary material for: Association between Mediterranean Dietary Pattern and Breakfast Quality with Physical Fitness in School Children: The HIIT Project
Source: Nutrients. 2021 Apr 18;13(4):1353. doi: 10.3390/nu13041353 (PMC8072945; doi:10.3390/nu13041353)
Supplement: Supplementary file 1 [file nutrients-13-01353-s001.zip › nutrients-1133447-supplementary.pdf]

Table S1. Pearson correlation between physical fitness and physical activity.

	Cardiorespiratory fitness	Upper-limbs muscular strength	Lower-limbs muscular strength	Speed–agility*
Moderate-to-vigorous physical activity	0.334**	0.026	0.245**	0.251**

Data were adjusted for sex. *In the speed–agility test, less time indicates a faster runner; a lower score indicates better performance. **p < 0.01
